# Genotype-associated core bacteria enhance host resistance against kiwifruit bacterial canker

**DOI:** 10.1093/hr/uhae236

**Published:** 2024-08-14

**Authors:** Min Fu, Yunhe Chen, Yong-Xin Liu, Xiaoxi Chang, Lei Zhang, Xinyi Yang, Li Li, Lixin Zhang

**Affiliations:** Anhui Province Key Laboratory of Integrated Pest Management on Crops, College of Plant Protection, Anhui Agricultural University, Hefei 230036, China; Key Laboratory of Agri-products Quality and Biosafety, Ministry of Education, Anhui Agricultural University, Hefei 230036, China; Anhui Province Key Laboratory of Integrated Pest Management on Crops, College of Plant Protection, Anhui Agricultural University, Hefei 230036, China; Key Laboratory of Agri-products Quality and Biosafety, Ministry of Education, Anhui Agricultural University, Hefei 230036, China; Shenzhen Branch, Guangdong Laboratory of Lingnan Modern Agriculture, Genome Analysis Laboratory of the Ministry of Agriculture and Rural Affairs, Agricultural Genomics Institute at Shenzhen, Chinese Academy of Agricultural Sciences, Shenzhen, Guangdong 518120, China; Anhui Province Key Laboratory of Integrated Pest Management on Crops, College of Plant Protection, Anhui Agricultural University, Hefei 230036, China; Key Laboratory of Agri-products Quality and Biosafety, Ministry of Education, Anhui Agricultural University, Hefei 230036, China; Anhui Province Key Laboratory of Integrated Pest Management on Crops, College of Plant Protection, Anhui Agricultural University, Hefei 230036, China; Key Laboratory of Agri-products Quality and Biosafety, Ministry of Education, Anhui Agricultural University, Hefei 230036, China; Anhui Province Key Laboratory of Integrated Pest Management on Crops, College of Plant Protection, Anhui Agricultural University, Hefei 230036, China; Key Laboratory of Agri-products Quality and Biosafety, Ministry of Education, Anhui Agricultural University, Hefei 230036, China; CAS Key Laboratory of Plant Germplasm Enhancement and Specialty Agriculture, CAS Engineering Laboratory for Kiwifruit Industrial Technology, Wuhan Botanical Garden, Chinese Academy of Sciences, Wuhan 430074, China; Anhui Province Key Laboratory of Integrated Pest Management on Crops, College of Plant Protection, Anhui Agricultural University, Hefei 230036, China; Key Laboratory of Agri-products Quality and Biosafety, Ministry of Education, Anhui Agricultural University, Hefei 230036, China

## Abstract

Both the phyllosphere and rhizosphere are inhabited by different kinds of microorganisms that are closely related to plant growth and health. However, it is not clear whether disease-resistant cultivars shape the microbiome to facilitate disease resistance. In this study, significant differences were found in the aboveground and belowground bacterial communities of disease-resistant and disease-susceptible cultivars grown in the same kiwifruit orchard. The phyllosphere of the resistant cultivar ‘Wanjin’ showed greater enrichment of *Pseudomonas* spp. and *Sphingomonas* spp. than the susceptible cultivar ‘Donghong’. The rhizosphere microbes of ‘Wanjin’ were less affected by field location, with significantly greater bacterial abundance than those of ‘Donghong’ and more bacteria with potential biocontrol properties. *Pseudomonas syringae* pv. *actinidiae* (*Psa*) infection significantly affected the microbiome of the phyllosphere of kiwifruit plants, especially that of ‘Donghong’. Resistant and susceptible kiwifruit cultivars exhibit distinct beneficial microbial recruitment strategies under *Psa* challenge. The phyllosphere of ‘Donghong’ in Jinzhai was enriched with *Sphingomonas* spp. and *Pantoea* spp. under *Psa* infection, while the rhizosphere of ‘Wanjin’ was enriched with *Sphingomonas* spp. and *Novosphingobium* spp. We further identified five key biomarkers within the microbial community associated with *Psa* infection. Inoculation experiments showed that *Lysobacter* sp. R34, *Stenotrophomonas* sp. R31, *Pseudomonas* sp. R10 and RS54, which were isolated from belowground compartments of ‘Wanjin’, could positively affect plant performance under *Psa* challenge. The combination use of *Pseudomonas* sp. R10 and *Stenotrophomonas* sp. R31 significantly improve the management of kiwifruit canker. Our findings provided novel insights into soil–microbe–plant interactions and the role of microbes in plant disease resistance and susceptibility.

## Introduction

Plants harbor many functionally distinct microorganisms in both the phyllosphere and rhizosphere. These microorganisms are considered to serve as an additional genome for plants and have coevolved with plants for more than 400 million years, resulting in the formation of a ‘holobiont’ [1, 2]. They interact with plants through reciprocity, symbiosis, and pathogenicity and play key roles in plant productivity, health, and ecology. Recently, the plant microbiome has been highlighted for its positive impact on host plant health and adaptation [3–7]. Nevertheless, to maximize the utilization of the plant microbiome to enhance plant disease resistance, it is imperative to elucidate the assembly, symbiotic patterns, and functions that govern the plant-associated microbiome [4].

The assembly of the plant microbiome is influenced by various biotic (e.g. host genotype, plant compartment, plant immune system) and abiotic (e.g. climate and soil type) factors [1]. Additionally, plant genotype and breeding also significantly affect the composition of the plant microbiome. For instance, 16S and ITS rRNA amplicon sequencing revealed that host niche, plant genotype and field geography significantly affect soybean endophytic bacterial and fungal communities [8]. Kwak *et al*. [9] reported that disease-resistant and disease-susceptible tomato varieties exhibited distinct rhizosphere microbial community structures, and transplanting the rhizosphere microbiota of disease-resistant plants suppressed disease symptoms in susceptible plants. A recent study revealed that disease-resistant cultivars had a more complex rhizobacterial community [10]. Furthermore, by integrating microbial community diversity sequencing, primary metabolism analysis, and genetics, it was shown that plants harboring a core microbiota in their spikes suppressed invasion by *Ustilaginoidea virens* by inhibiting branched-chain amino acid dependency [11], providing a sustainable pathway for the green prevention and control of foliar diseases.

Disease occurrence is also an important way to influence the assembly of the plant microbiome [12, 13]. As one of the most common sites of plant–microbe interactions, the root system has an important role in plant yield, nutrient absorption, and disease resistance. For example, Fusarium wilt disease affects bacterial and fungal communities in the below- and above-ground compartments of chili pepper plants, with a greater impact on the microbiome of the upper epidermis of stems and the inner layers of roots [4]. In addition, *Fusarium oxysporum* infection significantly altered the composition and gene expression of the root microbiome of common bean in a cultivar-dependent manner [14]. Accumulating evidence also suggests that plants are likely active participants in rhizosphere-mediated changes in the plant microbiome [1, 4]. Similar to the rhizosphere, the phyllosphere also serves as a crucial ecological niche for plants, playing a key role in plant disease resistance. By employing a multiomics approach, differences in the phyllosphere microbiota between non-infected citrus leaves and those infected with *Diaporthe citri* have been revealed [5]. However, there is still limited understanding regarding the structure and function of microbial communities in the phyllosphere, as well as how other economically valuable plants regulate their microbiomes under pathogen stress.

Kiwifruit (*Actinidia *Lindl.) is rich in a variety of vitamins and minerals and is known as the ‘king of fruits’ [15]. This plant originated in China, which is also its main distribution center, with the highest kiwifruit planting area and production worldwide. Statistical data for 2022 indicated that the kiwifruit cultivation area in China was 199 078 ha, yielding 2.38 MT of fruit, accounting for more than half of the total global kiwifruit production and planting area [16]. However, kiwifruit bacterial canker caused by *Pseudomonas syringae* pv. *actinidiae* (*Psa*) is a devastating disease that occurs in kiwifruit growing areas worldwide [17]. The infection spreads rapidly and is difficult to eradicate. It mainly affects the new shoots, branches, and leaves of the plant and can lead to cankers on the branches and trunks and leaf spots or even kill the plant, which has a major impact on kiwifruit yield and quality. Currently, the disease control measures rely on copper-based agents and agricultural antibiotics (such as kasugamycin, tetramycin, and zhongshengmycin), but the long-term application of these agents is not conducive to ecological sustainability [18, 19]. Therefore, there is an urgent need to find efficient and environmentally friendly methods to control this plant disease.

The plant microbiome is closely related to host disease resistance or susceptibility, and revealing how microbes and their host plants respond to diseases is important for advancing coevolutionary theories of plant-microbiome interactions [20]. Here we integrated amplicon sequencing, machine learning, and culture-dependent methods to investigate the impact of plant compartment, host genotype, field location, and *Psa* invasion on the kiwifruit microbiome and to compare changes in the microbiome between the aboveground and belowground compartments of *Psa*-infected resistant and susceptible kiwifruit cultivars under natural field conditions. We further identified key biomarkers in the microbial community associated with *Psa* infection. Subsequently, we screened and identified several key bacteria from the root endosphere and rhizosphere of *Psa*-resistant kiwifruit plants (‘Wanjin’) and explored their role in the control of kiwifruit bacterial canker.

## Results

### Impacts of plant compartment and genotype on kiwifruit microbiome

**Figure 1:**
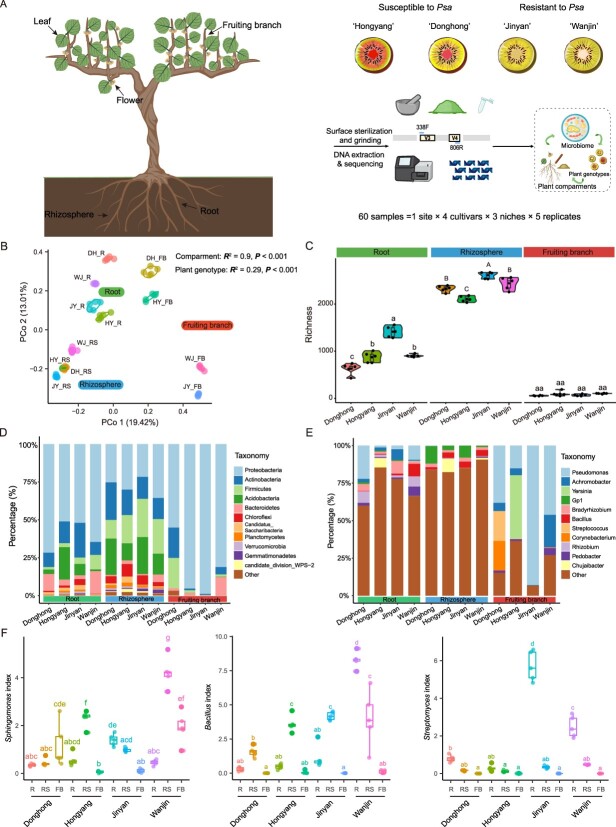
Community structure and microbial diversity in three ecological niches of four kiwifruit cultivars. **A** Workflow for processing, DNA extraction, and 16S rRNA sequencing of 60 samples from different ecological niches of four kiwifruit cultivars in Qianshan, Anhui Province. **B** Unconstrained PCoA (PCo1 and PCo2) with Bray–Curtis distances showing that the kiwifruit (cvs ‘Hongyang’, ‘Donghong’, ‘Jinyan’, and ‘Wanjin’) microbiomes from different ecological niches (fruiting branch endosphere, root endosphere, and rhizosphere) were separated on the first axis. **C** Richness indices of fruiting branch-, root-, and rhizosphere-associated bacteria for four plant cultivars. **D**, **E** Relative abundance of the top 12 dominant phyla (**D**) and dominant genera (**E**) in three ecological niches of different kiwifruit cultivars. **F** Box plots showing that the abundances of three bacterial genera (*Bacillus*, *Sphingomonas*, and *Streptomyces*) in the three ecological niches of the disease-resistant cultivar ‘Wanjin’ were significantly greater than those in the disease-susceptible cultivars (‘Donghong’ and ‘Hongyang’). The different letters represent significant differences (*P* < 0.05). R, root; RS, rhizosphere; FB, fruiting branch; HY, Hongyang; DH, Donghong; JY, Jinyan, WJ, Wanjin.

We first analyzed 16S rRNA gene amplicon sequences of bacterial communities in three niches of four cultivars of *Actinidia chinensis*, including two *Psa*-susceptible cultivars [‘Donghong’ (DH) and ‘Hongyang’ (HY)] and two *Psa*-resistant cultivars [‘Jinyan’ (JY) and ‘Wanjin’ (WJ)] [21, 22] (Fig. 1A). In total, 3 273 220 bacterial high-quality 16S rRNA reads were obtained from 60 samples (Supplementary Data Table S1). The reads were grouped into 7889 amplicon sequence variants (ASVs). The microbial communities were grouped mainly according to niche (*R*^2^ = 0.9, *P* < 0.001), followed by plant genotype (*R*^2^ = 0.29, *P* < 0.001; Fig. 1B, Supplementary Data Fig. S1A and B). However, significant differences were found in the bacterial communities on the fruiting branches of disease-resistant (JY and WJ) and disease-susceptible (DH and HY) cultivars, as evidenced by a significant deviation between these samples according to principal coordinate analysis (PCoA) (Fig. 1B). In addition, the bacterial α diversity differed significantly among niches, with the bacterial community of the rhizosphere being the most abundant, followed by those of the root endosphere and fruiting branch endosphere (Fig. 1C). In the rhizosphere, we found two resistant cultivars (JY and WJ) with significantly greater bacterial richness than HY (the most susceptible cultivar). The bacterial richness on the fruiting branches of the resistant and susceptible cultivars was similar (Fig. 1C), but the number of highly abundant bacteria was greater in WJ than in the two susceptible cultivars (Supplementary Data Fig. S1C).

At the phylum level, the root endosphere, rhizosphere, and fruiting branch endosphere were dominated by Proteobacteria, followed by Actinobacteria, Acidobacteria, Firmicutes, and Bacteroidetes (Fig. 1D). Notably, the microbial communities of the fruiting branch endosphere of HY and JY were represented mostly by Proteobacteria. However, at the genus level significant differences were found in the bacterial community composition among the three ecological niches, as evidenced by the greater relative abundance of the genera *Pseudomonas* and *Achromobacter* in the root endosphere and fruiting branch endosphere, whereas *Acidobacteria*_Gp1, *Bradyrhizobium* and *Bacillus* accounted for a greater proportion of the rhizosphere soil (Fig. 1E). Interestingly, the relative abundances of some genera with biocontrol potential, such as *Bacillus*, *Novosphingobium*, *Pseudomonas*, *Sphingomonas*, and *Streptomyces*, were significantly greater in disease-resistant cultivars, especially in the WJ cultivar (Fig. 1F, Supplementary Data Fig. S1D). The relative abundances of *Pseudomonas* spp. and *Sphingomonas* spp. in the fruiting branch endosphere of the WJ cultivar were greater than those in the fruiting branch endosphere of the two susceptible cultivars, whereas *Bacillus* spp. and *Streptomyces* spp. were more abundant in the root endosphere or rhizosphere (Fig. 1F, Supplementary Data Fig. S1D). In addition, *Achromobacter* spp., *Lysobacter* spp., *Paraburkholderia* spp., and *Pedobacter* spp. were more abundant in the root endosphere or rhizosphere of *Psa*-resistant cultivars than in those of *Psa*-susceptible cultivars (Fig. 1F, Supplementary Data Fig. S1D).

### Key bacterial taxa in the belowground compartment of ‘Wanjin’ are largely independent of geography

**Figure 2:**
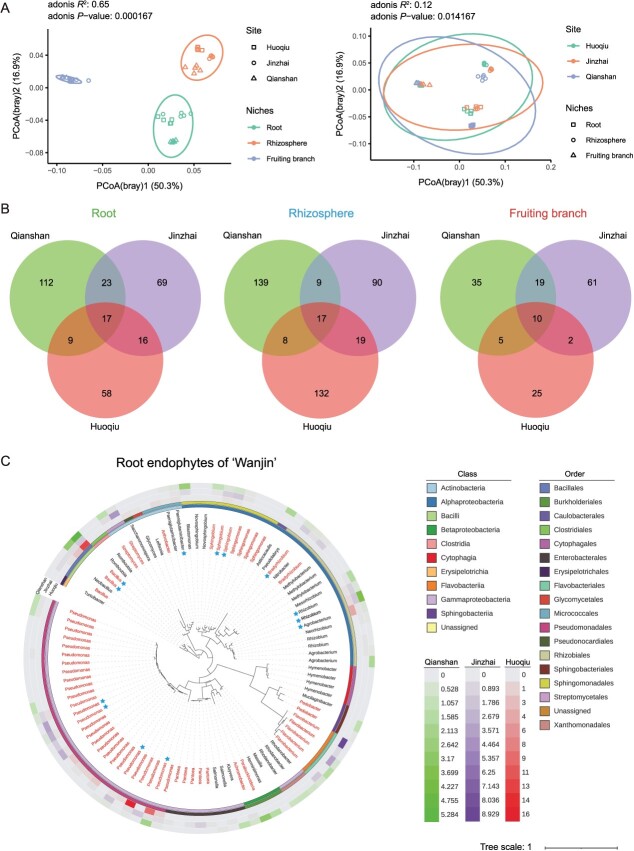
Effects of geographic location difference on microbiome assembly and diversity in three compartments of ‘Wanjin’ cultivar. **A** Unconstrained PCoA with Bray–Curtis distance was conducted to characterize the β diversity. **B** Common and specific high-abundance ASVs (with a relative abundance >0.1%) in the same ecological niche in ‘Wanjin’ from three different sites. **C** Phylogenetic trees showing the top 96 most abundant ASVs of bacteria in the roots of ‘Wanjin’ kiwifruit. The three outer rings represent the relative abundances of 96 ASVs present at Qianshan, Jinzhai, and Huoqiu. Bacterial genera labeled in red represent strains that could be isolated and cultured from ‘Wanjin’ kiwifruit plants. Bacterial ASVs labeled with blue pentagrams indicate core bacteria in the roots of ‘Wanjin’ kiwifruit plants.

To determine the effect of geographic location on kiwifruit bacterial communities, we further investigated the structure of the bacterial community of the WJ cultivar from three different counties (Huoqiu, Jinzhai, and Qianshan). A total of 2 628 809 bacterial high-quality 16S rRNA reads were obtained from 45 samples (Supplementary Data Table S2). The reads were grouped into 5149 ASVs. Bray–Curtis-based PCoA results showed that geographic location (*R*^2^ = 0.12, *P* < 0.05; Fig. 2A) affected the bacterial community structure but not as strongly as plant compartment (*R*^2^ = 0.65, *P* < 0.001; Fig. 2A). For bacterial communities, the effect of geographic location on the root endosphere (*R*^2^ = 0.88, *P* < 0.001) was less pronounced than that on the rhizosphere (*R*^2^ = 0.96, *P* < 0.001) and fruiting branch endosphere (*R*^2^ = 0.97, *P* < 0.001) (Supplementary Data Fig. S2A). Moreover, the effect of geographic location on the bacterial community was also evident in the richness (*P* < 0.001, Supplementary Data Fig. S2B) of the ASVs, with the bacterial abundance in the fruiting branch endosphere and root endosphere of Jinzhai kiwifruit being greater than that in the other two locations.

The three most highly abundant bacterial genera (*Bacillus*, *Acidobacteria*_Gp6, and *Sphingomonas*) in kiwifruit rhizosphere soils did not differ among the three regions (Supplementary Data Fig. S2C). However, the high abundance of bacteria on fruiting branches differed significantly among the three locations; for example, the genera *Salmonella* and *Pantoea* dominated in Huoqiu, *Agrobacterium* and *Novosphingobium* dominated in Jinzhai, and *Pseudomonas* and *Achromobacter* dominated in Qianshan (Supplementary Data Fig. S2C). It was found that *Pseudomonas* spp., *Bacillus* spp., and *Rhizobium* spp. (relative abundance ≥0.1%) were dominant in roots from the three sites, but there were differences in their abundances in the corresponding root endosphere (Supplementary Data Fig. S2C). In addition, we identified 10, 17, and 17 core (occurring at three locations with relative abundances >0.1%) bacterial taxa in the fruiting branch endosphere, root endosphere, and rhizosphere of WJ, respectively (Fig. 2B). Most of the core bacteria from the root endosphere and rhizosphere belonged to the genera *Acidobacteria*_Gp6, *Arthrobacter*, *Bacillus*, *Bradyrhizobium*, *Pseudomonas*, and *Streptomyces* (Fig. 2C, Supplementary Data Table S3). It is well known that the genera *Bacillus*, *Streptomyces*, and *Pseudomonas* contain many kinds of beneficial bacteria. The genus *Bradyrhizobium* contains biological nitrogen fixers. The 10 core bacteria in the fruiting branches belonged to eight genera, including *Pseudomonas*, *Achromobacter*, and *Sphingomonas* (Supplementary Data Table S3). The above results showed that geographical location had an effect on the bacterial communities of WJ but had little effect on the bacteria with high abundance in the belowground ecological niches of WJ.

### *Psa*-induced shifts in kiwifruit microbiome associated with plant genotypes

**Figure 3:**
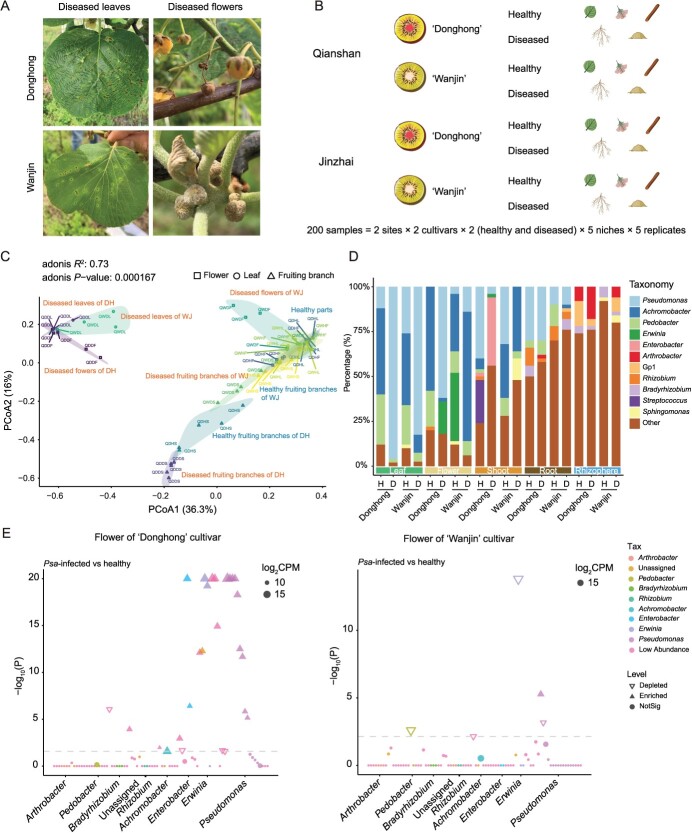
Effect of *Psa* infection on aboveground- and belowground-associated bacteria of susceptible and resistant kiwifruit plants. **A** Representative symptoms of kiwifruit bacterial canker on the leaves and flowers of ‘Wanjin’ and ‘Donghong’ kiwifruit in the field. **B** Samples were collected from healthy and diseased kiwifruit plants from kiwifruit orchards in Qianshan and Jinzhai, including leaves, flowers, fruiting branches, roots, and rhizosphere soils of the ‘Donghong’ and ‘Wanjin’ cultivars. **C** Unconstrained PCoA with Bray–Curtis distances showing the aboveground- and belowground-associated microbiota of the healthy and *Psa*-infected samples in Qianshan. **D** Relative abundances of the dominant genera in five niches of healthy and *Psa*-infected kiwifruit plants in Qianshan. H, healthy; D, diseased. **E** Manhattan plots showing ASVs depleted or enriched in flower samples infected with *Psa* versus healthy controls. ASVs are colored according to the bacterial genus.

To investigate the impact of *Psa* infection on the assembly of host bacterial communities and the differences between resistant and susceptible cultivars, we compared the bacterial community composition and diversity at five ecological niches in healthy and diseased plants of two kiwifruit cultivars, DH and WJ, in two different counties (Qianshan and Jinzhai) of Anhui Province (Fig. 3A and B). The results showed that *Psa* infection had a significant impact on bacterial diversity (e.g. on richness or Shannon index) in the aboveground parts of kiwifruit plants, while relatively little disturbance was observed in the underground ecological niches (i.e. root endosphere and rhizosphere) at both planting sites (Fig. 3C, Supplementary Data Figs S3A and S4A). In addition, *Psa* severely affected the composition of the bacterial community in the aboveground ecological niche of the DH cultivar compared with the WJ cultivar, regardless of the location of cultivation (Fig. 3D, Supplementary Data Fig. S4B). For example, in Qianshan the predominant bacteria in the healthy leaves and flowers of the two cultivars belonged to the genera *Pseudomonas*, *Acinetobacter*, and *Bacillus*. However, following the impact of the disease, the predominant microbial community in the leaf endosphere and flower endosphere of DH was composed primarily of *Pseudomonas*, whereas those of WJ remained largely unchanged (Fig. 3D). Notably, the trends in the diversity of ecological niches in the aboveground parts of kiwifruit after *Psa* infection in different regions were not entirely consistent, but the abundance of *Pseudomonas* spp. in the diseased plants significantly increased, and the microbial diversity of the ecological niche subsequently decreased (Fig. 3D, Supplementary Data Fig. S4B).

**Figure 4:**
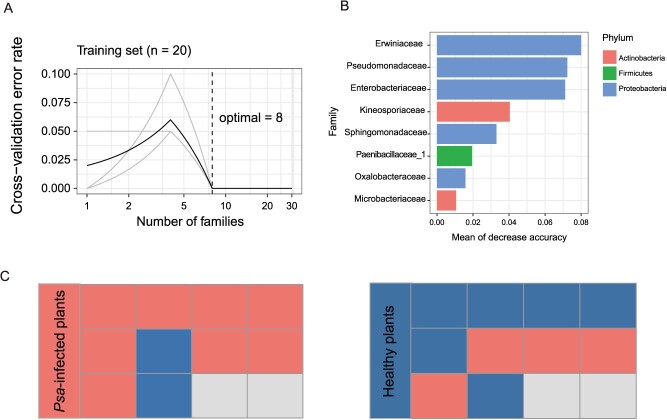
A random forest model detected bacterial taxa that could accurately predict *Psa* infection in kiwifruit plants. **A** The inset represents 10-fold cross-validation error as a function of the number of input families used to differentiate *Psa*-infected and healthy phyllosphere microbiota in order of variable importance. **B** A random forest approach was used to identify the top eight bacterial families that distinguish *Psa*-infected samples from healthy kiwifruit plants in Jinzhai. **C** Prediction of healthy and *Psa*-infected kiwifruit in Qianshan. Each square represents one of 10 healthy plants and 10 diseased plants. Current statuses of each plant are shown in the left panel, and the predicted plant states are shown in the right panel. Red indicates *Psa*-infected kiwifruit, and blue indicates healthy kiwifruit.

**Figure 5:**
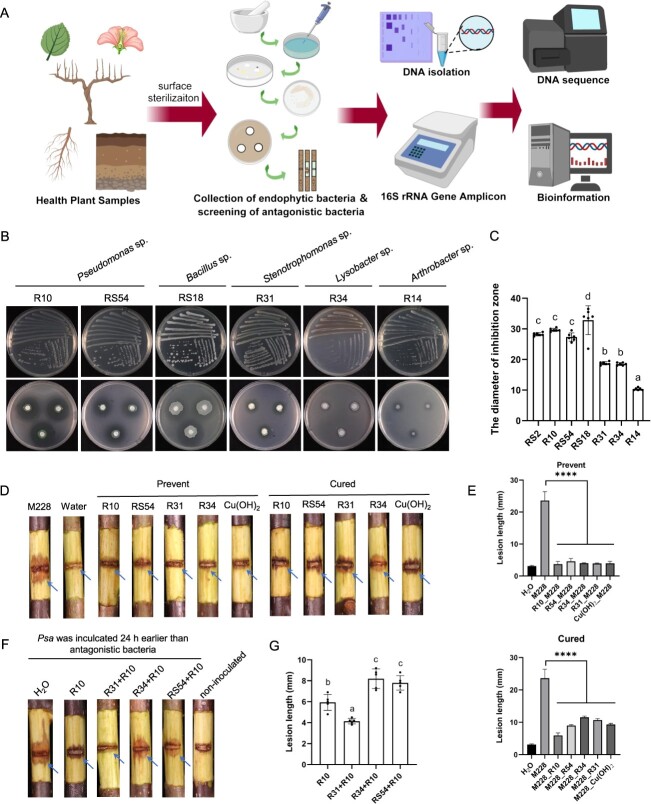
Bacteria from the root endosphere and rhizosphere of ‘Wanjin’ positively influenced plant resistance to *Psa* infection. **A** Workflow diagram for screening, characterization, and determination of the biocontrol efficacy of antagonistic bacteria from leaves, flowers, fruiting branches, roots, and rhizosphere soils of kiwifruit. **B**, **C** Antibacterial activity (**B**) and average diameter of the inhibition zone (**C**) of live cells of representative strains against *Psa* M228. **D** Protective ability and therapeutic activity of four antagonistic bacteria (*Pseudomonas* sp. R10, *Pseudomonas* sp. R54, *Stenotrophomonas* sp. R31, and *Lysobacter* sp. R34) against *Psa*-infected kiwifruit twigs. The symptoms were photographed at 21 days post-inoculation (dpi). **E** Lesion length on *Psa*-induced kiwifruit (‘Hongyang’) twigs after treatment with three antagonistic bacteria. The length of the lesion was measured after removing the bark at 21 dpi. R54_M228, inoculated with *Pseudomonas* sp. RS54 first, followed by *Psa* M228. M228_R54, inoculated with *Psa* M228 first, followed by *Pseudomonas* sp. RS54. ^****^P < 0.0001. **F** Therapeutic activity of functional groups of *Pseudomonas* sp. RS54–*Pseudomonas* sp. R10, *Pseudomonas* sp. R10–*Stenotrophomonas* sp. R31, and *Pseudomonas* sp. R10–*Lysobacter* sp. R34 against *Psa*-infected kiwifruit twigs. Symptoms were photographed at 21 dpi. **G** Lesion length on *Psa*-induced kiwifruit (‘Hongyang’) twigs after treatment with different synthetic microbial communities. The length of the lesion (after removing the bark) was measured at 21 dpi. Error bars indicate the standard deviations of the mean. The different letters represent significant differences (*P* < 0.05).

We compared the enriched and depleted ASVs (FDR-adjusted *P* < 0.05) in aboveground and belowground ecological niches of diseased and healthy plants for two cultivars in both regions. Manhattan plot analysis indicated that 7, 19, and 13 ASVs were enriched in the diseased leaves, flowers, and fruiting branches of the DH cultivar of Qianshan, respectively (Fig. 3E, Supplementary Data Fig. S3B). These ASVs were distributed in several genera, including *Achromobacter*, *Enterobacter*, *Erwinia*, and *Pseudomonas*. Among these genera, *Enterobacter* and *Erwinia* were the two with relatively high abundances in the phyllosphere, except for the genus *Pseudomonas*. For instance, in the flower endosphere, *Pseudomonas* spp. was the most abundant, accounting for 41% of the total, followed by *Enterobacter* spp. and *Erwinia* spp., which both accounted for 9% of the total abundance (Fig. 3E). In Jinzhai, ASVs in the diseased leaves and flowers of the DH cultivar were distributed in several genera, including *Pantoea*, *Sphingomonas*, and *Pseudomonas*. However, of the ASVs of WJ (Supplementary Data Fig. S4C), and the results were similar at the two different sites, indicating a relatively minor impact of *Psa* infection on the phyllosphere microbiome of WJ plants.

We further compared the community changes in the underground ecological niche between diseased and healthy kiwifruit plants. The results revealed that the root endosphere and rhizosphere of diseased DH plants from Qianshan were enriched with 38 and 25 ASVs, respectively, predominantly represented by *Achromobacter* spp., *Arthrobacter* spp., *Bradyrhizobium* spp., *Erwinia* spp., *Pedobacter* spp., and *Pseudomonas* spp. (Supplementary Data Fig. S3B). Similar bacterial genera were enriched in *Psa*-infected and healthy WJ plants, while *Arthrobacter* spp. and *Bradyrhizobium* spp. were significantly more highly enriched in the root endosphere or rhizosphere of WJ plants. In Jinzhai, ASVs enriched in diseased roots of DH were mainly distributed in the genera *Pantoea* and *Pseudomonas*, whereas ASVs significantly enriched in the rhizosphere soil were mainly represented by *Sphingomonas* spp. (Supplementary Data Fig. S4C). Moreover, the bacteria significantly enriched in the root endosphere and rhizosphere of *Psa*-infected WJ plants were *Sphingomonas* and *Novosphingobium* (Supplementary Data Fig. S4C), and, interestingly, similar results were observed in the rhizosphere of *Psa*-infected WJ plants in Qianshan.

### Biomarkers associated with *Psa* infection in kiwifruit

As mentioned above, *Psa* infection mainly affects the phyllosphere microbiome, especially that of leaves. Therefore, we investigated the occurrence of bacteria associated with *Psa* infection in the aboveground compartments using a machine learning-based random forest approach for identifying biomarkers between healthy and *Psa*-infected kiwifruit plants. We utilized the microbiome data of kiwifruit in Jinzhai to establish predictive models. Notably, the accuracy of discrimination at the family level reached 100% (Fig. 4A). In addition, biomarker contributions were evaluated using 10-fold cross-validation, which was repeated five times (Fig. 4A). The results showed that the cross-validation error curve stabilized when defining putative biomarker taxa using the eight most relevant bacterial taxa. These eight most relevant bacterial taxa were Erwiniaceae, Pseudomonadaceae, Enterobacteriaceae, Kineosporiaceae, Sphingomonadaceae, Paenibacillaceae_1, Oxalobacteraceae, and Microbacteriaceae (Fig. 4B). We further utilized kiwifruit samples from Qianshan as the testing set and applied a random forest model for prediction. The accuracy of the model was 70%, with a diseased status prediction accuracy of 80% and a healthy status prediction accuracy of 60% (Fig. 4C). Notably, bacteria other than *Psa* were frequently isolated from kiwifruit bacterial canker disease samples, most of which belonged to the families Erwiniaceae, Pseudomonadaceae, Enterobacteriaceae, Sphingomonadaceae, and Microbacteriaceae (M Fu, YH Chen, XX Chang, LX Zhang, unpublished data). Taken together, the results showed that these five families of bacteria may be key biomarker taxa for distinguishing *Psa*-infected plants from healthy plants.

### Bacteria from belowground compartment of ‘Wanjin’ positively affected plant performance against *Psa* infection

To determine the disease-suppressive capacity of the microbiota in disease-resistant cultivars, we isolated culturable bacteria from the fruiting branches, roots, and rhizosphere soils of WJ kiwifruit originating from Jinzhai (Fig. 5A). In total, 420 bacterial strains were obtained on nutrient agar medium. Of these, 58 (13.8%) strains exhibited significant antagonistic activity against *Psa*. Among them, 18 strains were obtained from aboveground parts (10 strains from leaves and 4 strains from flowers), while 40 strains were obtained from underground parts (16 strains from roots and 24 strains from rhizosphere). Notably, 14 strains exhibited inhibition zones exceeding 10 mm in size (Fig. 5B and C), suggesting that they may be effective in controlling bacterial canker of kiwifruit. These strains with biocontrol potential were taxonomically classified into nine bacterial genera: *Achromobacter*, *Arthrobacter*, *Bacillus*, *Delftia*, *Lysobacter*, *Paenibacillus*, *Pedobacter*, *Pseudomonas*, and *Stenotrophomonas* (Supplementary Data Fig. S5). Importantly, *Pseudomonas* sp. RS2, *Pseudomonas* sp. R10, *Pseudomonas* sp. RS54, and *Bacillus* sp. R18 exhibited strong antagonism against *Psa*, with average diameters of the inhibition zones 28.3, 29.7, 27.3, and 32.8 mm, respectively (Fig. 5C).

We further selected four representative strains (*Pseudomonas* sp. R10, *Pseudomonas* sp. RS54, *Stenotrophomonas* sp. R31, and *Lysobacter* sp. R34) with inhibitory activity to test their biocontrol efficacy against *Psa* on detached twigs of HY. The representative strains were inoculated 24 h prior to *Psa* inoculation to investigate the preventive effect. The results indicated that the lesions on the twigs treated with strains with biocontrol potential were significantly smaller than those on the twigs treated with H_2_O, with an average lesion diameter of 3.7–4.6 mm (Fig. 5D and E). We also evaluated the control effect of four biocontrol potential strains. The results showed that strain R10 exhibited superior therapeutic efficacy compared with Cu(OH)_2_ and the other two strains (R31 and R34), with average lesion lengths and treatment efficacy measures of 5.9 mm and 75% for R10, respectively (Fig. 5D and E).

The roots and rhizosphere soils of WJ kiwifruit are rich in a variety of beneficial microorganisms that antagonize *Psa*, with a high proportion of *Pseudomonas* spp. exhibiting biocontrol potential. To determine whether any other bacteria could cooperate with *Pseudomonas* sp. to resist *Psa* infection, we constructed three hybrid biocontrol agents, namely *Pseudomonas*–*Pseudomonas* (PP), *Pseudomonas*–*Stenotrophomonas* (PS), and *Pseudomona*s–*Lysobacter* (PL). Prior to constructing the hybrid biocontrol agents, compatibility tests were conducted on two members of the hybrid biocontrol agents. The results indicated that *Pseudomonas* sp. R10 and *Stenotrophomonas* sp. R31 exhibited favorable compatibility, while *Pseudomonas* sp. R10 had weak antagonistic activity against *Pseudomonas* sp. R54 and *Lysobacter* sp. R34 (Supplementary Data Fig. S6). The therapeutic effect of three hybrid biocontrol agents was further evaluated (Fig. 5F and G). The diameter of lesions on PS-treated twigs (inoculated with *Psa* 24 h before hybrid biocontrol agent inoculation) was significantly smaller (4.1 mm) than that on branches treated with PP (7.8 mm) or PL (8.2 mm) (Fig. 5F and G). Besides, compared with antagonistic bacteria alone, the hybrid biocontrol agent PS enhanced the therapeutic effect against kiwifruit bacterial canker by 30.7% (PS versus *Pseudomonas* sp. R10) and 61.3% (PS vs *Stenotrophomonas* sp. R31), respectively. Taking these results together, the synergistic effect of *Pseudomonas* sp. R10 and *Stenotrophomonas* sp. R31 could greatly improve the control of kiwifruit bacterial canker.

## Discussion

The plant microbiome is closely linked to plant growth and disease resistance and is important for community stabilization, maintenance of host biodiversity, and ecosystem function [3, 5, 6, 9, 11, 13, 23, 24]. However, there is still a lack of understanding regarding how the cooperation among members of the plant-associated microbial community influences microbial community establishment and plant health. In this study, we analyzed the impact of host genotype, ecological niche, and geographic location on the kiwifruit microbiome and explored the changes in the phyllosphere and rhizosphere microbiomes between *Psa*-infected and healthy kiwifruit plants.

The microbial communities on plants are influenced by various factors, such as soil type, plant compartment, plant development, host genotype, plant immune system, and season [1, 25]. Accumulating studies on common bean [14], soybean [8], and chili pepper [4] have shown that ecological niches have a greater impact on microbial diversity and composition than disease occurrence and plant genotype. For example, the plant compartment had the greatest influence on the total microbiome of chili pepper, followed by wilt disease and sampling location [4]. Here, we found that the plant compartment had the greatest influence on the kiwifruit microbiome, followed by the host genotype. In addition, the bacterial composition at the genus levels (Supplementary Data Fig. S1E) also showed that plant compartment is highly selective for microbial communities. During plant interactions with various microorganisms, different parts of the plant selectively attract beneficial microbes to promote their survival and adaptation to environmental stresses.

Recent reports have shown that host genotype also plays a key role in shaping the plant microbiome [26]. For example, indica rice and japonica rice form distinct root microbiomes, serving as potential biomarkers for differentiation between the two rice varieties [27]. In a study of the soybean microbiome, the host genotype explained more variations in stem and leaf microbial community composition [8]. Besides, it was found that *Fusarium oxysporum*-resistant cultivars exhibited higher bacterial diversity in the rhizosphere soils and were enriched with beneficial taxa such as *Pseudomonas* spp. and *Bacillus* spp. [28]. In this study, we found that both the root endosphere and rhizosphere bacterial diversity were greater in the resistant cultivars than in the two susceptible cultivars, and the composition of fruiting branch endophytes significantly differed between the resistant and susceptible cultivars. More bacteria with biocontrol potential, such as *Achromobacter*, *Bacillus*, *Lysobacter*, *Pedobacter*, *Rhizobium*, and *Streptomyces*, were enriched in WJ cultivars that showed better resistance in the field. All these data suggest that disease-resistant cultivars exhibit greater microbial abundance in their underground ecological niche, with more intricate interactions among microorganisms and greater diversity of disease-resistant microbial communities. However, it remains unclear whether disease-resistant cultivars have shaped a microbiota capable of resisting disease or whether disease-resistant microbiota have enhanced the resistance of the cultivars. It has been suggested that resistance breeding in plants may inadvertently co-select for plant traits that affect the recruitment of microbiota specific to beneficial cultivars [14, 28]. Several previous studies have also shown that the domestication of plants exerts selection pressure on plant-associated microorganisms [29, 30]. In summary, in both natural and artificial selection, plant evolution has exerted significant selective pressure on the rhizosphere microbiome [7, 31].

In addition to plant compartments and host genotypes, pathogen invasion is another important biotic factor affecting plant microbiome assembly. Previous studies have shown that the microbiomes of a variety of plants (e.g. soybean, pepper, citrus, and tomato) undergo significant changes under pathogen challenge [4, 5, 11, 13, 14]. In this study, *Psa* infection significantly reduced the microbial community diversity in the kiwifruit aboveground compartments and altered its community structure, with these changes being particularly pronounced in the aboveground compartments of the DH cultivar. Currently, there are various competitive interactions between plant microorganisms, such as resource competition, secretion of antimicrobial compounds, contact-dependent competition, and production of volatile organic compounds, all of which affect the structure and diversity of microbial communities [1, 32]. Besides, some pathogens can influence the recruitment and colonization of host microbiomes. It has been reported that the effectors VdAve1 and VdAMP2 secreted by the fungus *Verticillium dahliae* could manipulate the microbial community in the soil environment [33]. Bacteria have also evolved a variety of weapons to harm and kill competitors, such as Type IV secretion systems (T4SS), T6SS, T7SS, nanotubes, and small-molecule toxins [34]. Many Gram-negative bacteria possess T6SS, which serve as a contact-dependent weapon for the bacteria to transport effectors and kill target cells [35]. Previous studies have shown that *Burkholderia rhizoxinica* could utilize T3SS to regulate its symbiosis efficiency with *Rhizopus microsporus* [36]. Notably, T3SS and T6SS play important roles in *Psa* invasion of kiwifruit [37, 38]. It is hypothesized that Psa may utilize T3SS or T6SS to deliver effectors to bacterial and eukaryotic target cells, thereby gaining a competitive ecological advantage in its habitat.

After infection, *Psa* usually directly displaces some low-abundance communities at infection sites and becomes predominant as the disease progresses. During this process, pathogen-infected plants can rescue or protect their offspring by attracting beneficial microorganisms, either directly or indirectly, in what is often referred to as the ‘cry for help’ strategy [20]. Accumulating evidence suggests that the roots of multiple plants (e.g. *Arabidopsis thaliana*, chili peppers, and wheat) possess qualities that allow plants to recruit beneficial microorganisms [4, 39–41]. A recent study revealed that citrus leaves infected with *Diaporthe citri* were enriched with *Sphingomonas* spp. and several new microbes, which provides further evidence of the plant’s ‘cry for help’ strategy in the phyllosphere [5]. Here, we also observed similar phenomena. For example, the aboveground compartment of DH in Jinzhai was significantly enriched with *Sphingomonas* spp., *Pantoea* spp., and *Hymenobacter* spp. after infection with *Psa*. The root endosphere and rhizosphere of *Psa*-infected WJ kiwifruit plants exhibited significant specific enrichment of *Sphingomonas* spp. and *Novosphingobium* spp. Many of these species of *Sphingomonas* and *Pantoea* have been shown to have good biocontrol value [5, 23]. Interestingly, we found that the intensity of recruitment of beneficial microorganisms to the aboveground compartments of disease-susceptible cultivars appeared to be greater than that of disease-resistant cultivars. This phenomenon is understandable, as our earlier results indicated that the phyllosphere and rhizosphere of a disease-resistant cultivar of WJ was inhabited by many bacteria with biocontrol potential under healthy conditions and were comparatively less influenced by external environmental factors. These biocontrol resources in disease-resistant cultivars may be depleted when battling exotic invaders such as *Psa*, but this approach may be quicker and more effective than in susceptible cultivars (in which recruitment of biocontrol resources begins after *Psa* invasion). It has been suggested that several members of the resident microbiome may also be potential pathogens, and their increased abundance or manipulation by pathogenic bacteria to form a pathobiome may exacerbate disease development [42, 43]. This is analogous to the medical term ‘syndemic’, referring to the synergistic effects between diseases [44]. In this study, *Erwinia* spp. were enriched on both the leaves and fruiting branches of diseased DH plants in Qianshan. Notably, many species of *Erwinia* are recognized as prevalent plant pathogens. This highlights the significance of field management, as regular removal of diseased plant residues helps reduce potential pathogens in both aboveground and belowground ecological niches, while beneficial agricultural activities such as fertilization aid in attracting more beneficial resources to the plant rhizosphere.

The rhizosphere is home to many beneficial microorganisms that play a key role in regulating plant growth and health [3]. Additionally, rhizosphere microorganisms serve as a seed bank for phyllosphere microorganisms. To date, many biocontrol resources have been screened in the rhizosphere or plant endosphere, with *Bacillus* spp., *Streptomyces* spp., and *Pseudomonas* spp. being the most popular biocontrol agents [45, 46]. A growing body of literature has demonstrated the efficacy of *Bacillus velezensis* WL–23 and *Paenibacillus polymyxa* YLC1 in controlling kiwifruit bacterial canker caused by *Psa* [18, 46]. In this study, the underground parts of WJ exhibited significant amounts of potential biocontrol resources, with a few bacteria playing a crucial role in controlling kiwifruit bacterial canker disease, such as *Bacillus* sp. RS18, *Lysobacter* sp. R34, *Pseudomonas* sp. RS54, and *Stenotrophomonas* sp. R31. Among them, *Pseudomonas* spp. accounted for a high percentage of antagonistic bacteria, and most of them had good antagonistic activities. A previous study on kiwifruit rhizosphere microorganisms showed similar findings [47]. It is hypothesized that bacteria belonging to the genus *Pseudomonas* may exhibit a preference for the kiwifruit host. This is further supported by the predominance of *Pseudomonas* species in the endophytic microbiome of healthy kiwifruit leaves, flowers, and fruiting branches. Several previous studies have also shown that members of the plant-associated microbiome also compete directly or indirectly with members of closely related microbiomes [1, 48]. In the present study, we also investigated the ability of different antagonistic resources to co-control disease development. The combined use of two different *Pseudomonas* species was less effective than the use of two *Pseudomonas* species alone, thus confirming the aforementioned perspective.

In conclusion, our study revealed differences in the phyllosphere and rhizosphere microbiomes of disease-resistant and disease-susceptible cultivars under healthy conditions and explored potential echoing strategies in disease-resistant and disease-susceptible cultivars of kiwifruit following *Psa* infection. This study contributes useful information for understanding the composition and drivers of phyllosphere and rhizosphere microbes in kiwifruit, as well as the interactions among disease resistance, endophytic microbes, and plant pathogens.

## Materials and methods

### Sampling

To assess the effects of host genotype and ecological niche on the kiwifruit microbiome, leaf, flower, fruiting branch, root, and rhizosphere soil samples of four healthy kiwifruit cultivars (‘Hongyang’, ‘Donghong’, ‘Jinyan’ and ‘Wanjin’) were collected in Qianshan city (30°57′N, 116°42′E; altitude 76 m) of Anhui Province, China, in April 2023 (Supplementary Data Table S1). The soil type in the field is sandy soil. ‘Hongyang’ (HY) and ‘Donghong’ (DH) are both red-fleshed kiwifruit cultivars of *Actinidia chinensis*, with HY being highly susceptible to kiwifruit bacterial canker disease, and DH, a recent selection from the *F*_1_ offspring of HY, also showing susceptibility to kiwifruit bacterial canker according to field surveys and laboratory assessments [22, 49]. ‘Jinyan’ (JY) and ‘Wanjin’ (WJ) are both yellow-fleshed kiwifruit cultivars of *A. chinensis* with moderate resistance to kiwifruit bacterial canker [21, 22]. In the spring of 2023, the incidence rates of diseases on the canes of HY, DH, JY, and WJ were 46.7%, 23.3%, 10%, and 3.3%, respectively. In each orchard, kiwifruit plants that did not show symptoms (e.g. necrosis of branches and trunks, leaf spots, flower rot) of kiwifruit bacterial canker and from which *Psa* was not isolated were categorized as healthy kiwifruit plants. Conversely, plants that showed kiwifruit bacterial canker symptoms and from which *Psa* was isolated from diseased parts were categorized as *Psa*-infected plants. Briefly, five healthy kiwifruit plants were randomly selected from each cultivar as five biological replicates. Leaf, flower, and fruiting branch samples were collected from healthy kiwifruit using sterile scissors. Approximately 1–3 mm of the surface of the roots was selected as the rhizosphere soil. Each replicate sample was a composite consisting of a mixture of five individual samples. In total, 100 experimental treatments (4 host genotypes × 5 plant compartments × 5 replicates) were generated (Supplementary Data Table S1).

To determine the effects of sampling site on the aboveground and belowground microbiota, leaf, flower, fruiting branch, root, and rhizosphere soil samples of healthy WJ kiwifruit were collected in Qianshan city (30°57′N, 116°42′E; altitude 76 m), Huoqiu county (32°09′N, 116°17′E; altitude 33.2 m) and Jinzhai county (31°76′N, 115°85′E; altitude 67.3 m) of Anhui province in April 2023. The three sites have a subtropical monsoon climate, with Qianshan and Jinzhai located in the southern and northern regions of the Ta-pieh Mountains, respectively. The soil types of the fields in Huoqiu and Jinzhai are natural brown soil and sandy soil, respectively. The sampling methods were the same as those described above. In total, 75 experimental treatments (3 field locations × 5 plant compartments × 5 replicates) were generated (Supplementary Data Table S2).

To determine the impact of kiwifruit bacterial canker on the kiwifruit microbiome, leaf, flower, fruiting branch, root, and rhizosphere soil samples were collected from healthy plants and plants infected with *Psa* in Qianshan city and Jinzhai county. The sampling method for healthy samples has been described previously. For the *Psa*-infected samples, five kiwifruit trees with typical symptoms of kiwifruit bacterial canker were randomly selected as diseased trees, and diseased leaves, flowers, fruiting branches, roots, and rhizosphere soils were collected in the same way. A total of 200 samples [2 field locations × 2 cultivars × 2 (healthy and diseased) × 5 niches × 5 replicates] were generated (Supplementary Data Tables S4 and S5).

### DNA extraction and amplicon sequencing

The removal of epiphytic microorganisms from the samples was performed according to Yang *et al*. [8]. DNA from each sample was extracted using the E.Z.N.A.^®^ Soil DNA Kit (Omega Bio-tek, Norcross, GA, USA) according to the manufacturer’s instructions. The V3–V4 region of the bacterial 16S rRNA gene was amplified using the primers 338F/806R (Supplementary Data Table S6). The amplicons were sequenced on an Illumina Nextseq 2000 at Majorbio Bio-Pharm Technology Co. Ltd (Shanghai, China).

### Bioinformatics analysis

Analysis of sequencing amplicon data was conducted in R version 4.3.0 using the EasyAmplicon pipeline v1.19 [50]. Briefly, the 16S rRNA reads were processed for merging (—fastq_mergepairs), quality filtering, and ASV dereplication (—derep_fulllength) using VSEARCH v2.22.1 [51]. The -unoise3 command in USEARCH v10.0 was used to denoise (chimeras were removed) non-redundant sequences [52]. The feature table was created by VSEARCH. Taxonomic assignment was performed via the RDP database (rdp_16s_v18). Analysis of α and β diversity, high-abundance microbiome screening, species annotation classification, and reference quantitative feature table output analysis were performed in R v4.3.0 using the EasyAmplicon pipeline v1.19. [50, 53]. A Venn diagram was constructed with EVenn [54]. Practical boxplot analysis and PCoA were conducted with ImageGP [55]. Multiple sequence alignment and phylogenetic analysis were conducted using MUSCLE v3.8.31 [56] and IQ-TREE v1.6.10 (maximum likelihood method), respectively. The phylogenetic tree was visualized in iTOL v6 [57]. Core ASVs were defined based on their persistent occurrence with high abundance (relative abundance >0.1%) in the plant compartments [58]. Biomarker prediction was based on the approach outlined in Zhang *et al*. [27]. Data from kiwifruit in Jinzhai county were utilized as a training set, and the randomForest function was employed to develop classification models for healthy plants and *Psa*-infected plants. The test set used for the model was based on healthy and diseased kiwifruit in Qianshan city with default parameters.

### Isolation and identification of culturable bacteria

Endophytic bacterial isolation was performed on leaf, flower, and fruit branch samples of WJ kiwifruit collected from kiwifruit orchards in Jinzhai County. Briefly, the samples were sterilized with 75% ethanol for 30–45 s and then rinsed with sterilized water. After sterilization, the samples were cut into small pieces, ground thoroughly and soaked in sterilized water for 5 min. The grinding solution was diluted and an aliquot (30 μl) of each dilution was plated on nutrient agar plates and incubated at 28°C for 2–7 days. The isolation of bacteria from the soil samples followed the methodology outlined by Yan *et al*. [47]. Single colonies were picked based on morphological characteristics (smoothness, size, color, etc.).

Bacterial DNA was extracted according to a previously described method [59]. The 16S rRNA gene was amplified with the PCR primers 27F and 1492R (Supplementary Data Table S6) and sequenced by Sanger sequencing in both orientations. Multiple alignments were conducted utilizing MAFFT (https://mafft.cbrc.jp/alignment/server/index.html). The phylogenetic tree was constructed by the maximum likelihood method in MEGA 7 [60].

### *In vitro* antagonistic activities of bacteria against *Psa*

A strongly pathogenic bacterial strain, *Psa* M228 [38], was used as the target bacterium for screening potential antagonistic bacteria. *Psa* M228 was transferred to King’s B (KB) liquid medium for 24 h of shaking culture (28°C, 180 rpm) and its OD_600_ value was adjusted to 2.0 [47]. Then 5 ml of *Psa* suspension was transferred into melted KB agar medium and poured into sterile Petri dishes. The tested bacterial isolates were spot-inoculated onto KB agar medium and incubated at 28°C for 2 days.

### Biocontrol effects of antagonistic bacteria

To explore the ability of antagonistic bacteria to protect against attack by *Psa*, representative bacteria were applied to twigs of kiwifruit plants (HY) 24 h before inoculation with *Psa* M228. Sterilization and other treatments of kiwifruit branches were performed according to previous methods [18]. A razor blade was used to make a wound in the center of the twigs to the xylem. An aliquot of 10 μl of bacterial fermentation solution (OD_600_ = 0.1) was dropped onto the wound site. At 24 h post-inoculation, *Psa *culture solution (10 μl, OD_600_ = 0.1) was administered to the same wound site. The inoculated twigs were put onto the trays and then placed in a mist chamber at 90% relative humidity. For testing its control effect against kiwifruit bacterial canker, an aliquot of 10 μl of *Psa* culture (OD_600_ = 0.1) was dropped onto the wound site and kept in a mist chamber at 90% relative humidity for 24 h. Then, 10 μl of test bacteria was dropped onto the same wound site. The lesion lengths (bark removed) were measured at 21 days post-inoculation (dpi).

### Assembly of hybrid biocontrol agents and their biocontrol efficacy against kiwifruit bacterial canker

The assessment of the compatibility between two members of the hybrid biocontrol agents and the assembly of hybrid biocontrol agents were conducted following the procedure outlined by Lin *et al*. [61]. Strains R10, R31, R34, and RS54 were individually cultured at 28°C for 12 h, and their OD_600_ values were adjusted to 1.0. Subsequently, a cell suspension of strain R10 was mixed in a 1:1 ratio with sterile water and with cell suspensions of strains RS54, R31, and R34. Inoculation of *Psa* M288 onto twigs was conducted following the methods outlined above. Twenty-four hours post-inoculation with *Psa* M288, 10 μl of mixtures of hybrid biocontrol agent was applied to the wounds on kiwifruit twigs. Twigs inoculated with sterile water served as non-inoculated controls. The inoculated twigs were kept in a mist chamber at 90% relative humidity.

### Statistical analysis

The statistical analysis of α diversity was conducted using ANOVA with *P* values and *post hoc* tests with Tukey’s HSD. PERMANOVA was performed using vegan’s function ‘adonis()’ to measure effect size and significance on β diversity. Statistical analysis of the antibacterial activity of live cells of bacterial strains was conducted with IBM SPSS Statistics 21.0 (IBM Corp., Armonk, NY) using one-way ANOVA. Means were separated using Duncan’s test at a significance level of *P* = 0.05. Data were graphed using Prism 8.2.1 (GraphPad, CA, USA) to determine the length of lesions in the pathogenicity test. Data from the two groups were compared using Student’s *t*-test (unpaired).

## Supplementary Material

Web_Material_uhae236

## Data Availability

The 16S sequencing raw data have been deposited at the National Center for Biotechnology Information (NCBI) under the accession number PRJNA1109462.
