# Supplementary material for: Genotype-associated core bacteria enhance host resistance against kiwifruit bacterial canker
Source: Hortic Res. 2024 Aug 14;11(11):uhae236. doi: 10.1093/hr/uhae236 (PMC11539023; doi:10.1093/hr/uhae236)

**Genotype-associated core bacteria enhance host resistance against kiwifruit bacterial canker**

Min Fu^1,2#^, Yunhe Chen^1,2#^, Yong-Xin Liu^3^, Xiaoxi Chang^1,2^, Lei Zhang^1,2^, Xinyi Yang^1,2^, Li Li^4^, Lixin Zhang^1,2^*

^1^ Anhui Province Key Laboratory of Integrated Pest Management on Crops, College of Plant Protection, Anhui Agricultural University, Hefei 230036, China

^2^ Key Laboratory of Agri-products Quality and Biosafety, Ministry of Education, Anhui Agricultural University, Hefei 230036, China

^3^ Shenzhen Branch, Guangdong Laboratory of Lingnan Modern Agriculture, Genome Analysis Laboratory of the Ministry of Agriculture and Rural Affairs, Agricultural Genomics Institute at Shenzhen, Chinese Academy of Agricultural Sciences, Shenzhen, Guangdong 518120, China

^4^ CAS Key Laboratory of Plant Germplasm Enhancement and Specialty Agriculture, CAS Engineering Laboratory for Kiwifruit Industrial Technology, Wuhan Botanical Garden, Chinese Academy of Sciences, Wuhan 430074, China

* **Corresponding author**

**Lixin Zhang**

Tel: 86-0551-65786312, Fax: 86-0551-65786321, E-mail: [lxzhang@ahau.edu.cn](mailto:lxzhang@ahau.edu.cn)

^#^ These authors have contributed equally to this work

**Running title**: Core bacteria enhance disease resistance in kiwifruit

**E-mails**

Min Fu: [fumin2022@ahau.edu.cn](mailto:fumin2022@ahau.edu.cn)

Yunhe Chen: [Cyh202206@stu.ahau.edu.cn](mailto:Cyh202206@stu.ahau.edu.cn)

Yong-Xin Liu: [liuyongxin@caas.cn](mailto:liuyongxin@caas.cn)

Xiaoxi Chang: [changxiaoxi@stu.ahau.edu.cn](mailto:changxiaoxi@stu.ahau.edu.cn)

Lei Zhang: [lzhang1318@stu.ahau.edu.cn](mailto:lzhang1318@stu.ahau.edu.cn)

Xinyi Yang: [YXY1@stu.ahau.edu.cn](mailto:YXY1@stu.ahau.edu.cn)

Li Li: [lili@wbgcas.cn](mailto:lili@wbgcas.cn)

Lixin Zhang: [lxzhang@ahau.edu.cn](mailto:lxzhang@ahau.edu.cn)

**ORCID**

**Min Fu**: 0000-0002-4291-7664

**Yong-Xin Liu**: 0000-0003-1832-9835

**Lixin Zhang**: 0000-0002-2163-7655

**Supplementary Figure 1: Host niche and genotype impacts on kiwifruit microbiome composition. A, B**. Unconstrained PCoA (PCo1 and PCo2) with Bray-Curtis distance was conducted to characterize the beta diversity and analyzed in PERMANOVA to test for differences. The microbial communities were grouped mainly according to niches (*R*^2^ = 0.9, *P* < 0.001), followed by plant genotypes (*R*^2^ = 0.29, *P* < 0.001). **C**. Common and specific high abundance of ASVs in four cultivars in the same ecological niche. **D**. Box plots showed that the abundance of six bacterial genera in three ecological niches of the disease-resistant cultivar ‘Wanjin’ was significantly higher than that of the disease-susceptible cultivars (‘Donghong’ and ‘Hongyang’). The different letters represent significant differences (*p* < 0.05, ANOVA, Tukey HSD test).


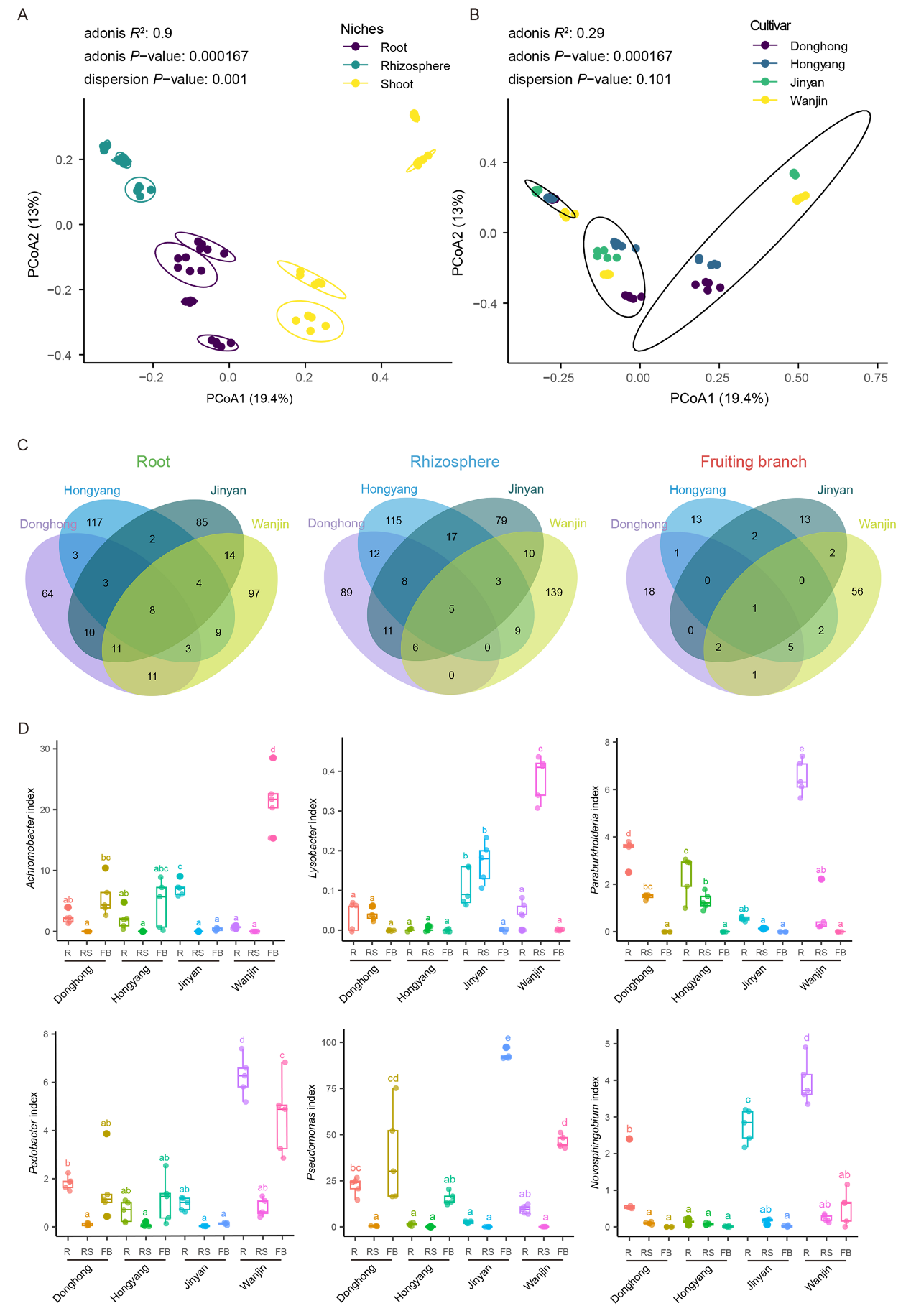


**Supplementary Figure 2: Influence of geographic location on bacterial community composition in ‘Wanjin’ root, rhizosphere, and fruiting branch. A.** Unconstrained PCoA (PCo1 and PCo2) with Bray-Curtis distance was conducted to characterize the beta diversity and analyzed in PERMANOVA to test for differences. The effect of geographic location on root (*R*^2^ = 0.88, *P* < 0.001) was less compared to rhizosphere (*R*^2^ = 0.96, *P* < 0.001) and fruiting branch (*R*^2^ = 0.97, *P* < 0.001). **B**. α-diversity of bacterial communities was estimated by Richness index. Different letters indicate significant differences among indicated results (ANOVA and Tukey’s HSD, *P*<0.05). Boxes represent the 75th percentile (upper) and 25th percentile (lower). Upper and lower whiskers refer to data not more than 1.5 times the interquartile spacing from the upper and lower edges of the box, respectively. **C**. Relative abundance of dominant genus in three ecological niches of ‘Wanjin’ kiwifruit at three sites (Qianshan City, Jinzhai County, Huoqiu County). The different letters represent significant differences (*p* < 0.05, ANOVA, Tukey HSD test).


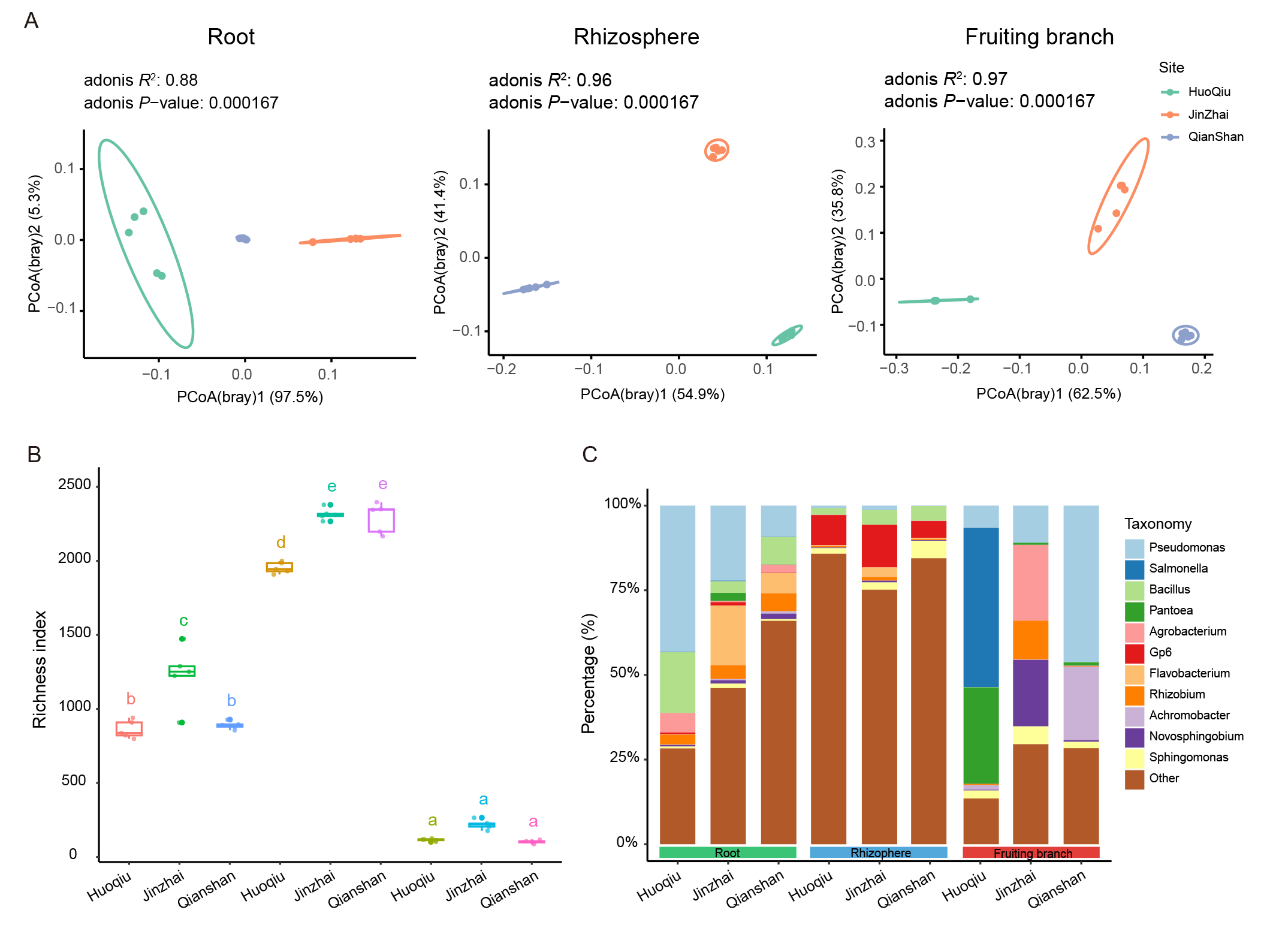


**Supplementary Figure 3: Effect of *Psa* infection on phyllosphere and rhizosphere associated bacteria of susceptible and resistant kiwifruit plants in Qianshan City. A.** Richness index and Shannon index of bacteria associated with fruiting branches, roots, and rhizosphere of healthy and *Psa*-infected kiwifruit plants in Qianshan City. **B**. Manhattan plots showing ASVs depleted or enriched in samples infected with *Psa* vs healthy samples in Qianshan city. ASVs were arranged and colored according to the bacterial genus. The different letters represent significant differences (*p* < 0.05, ANOVA, Tukey HSD test).


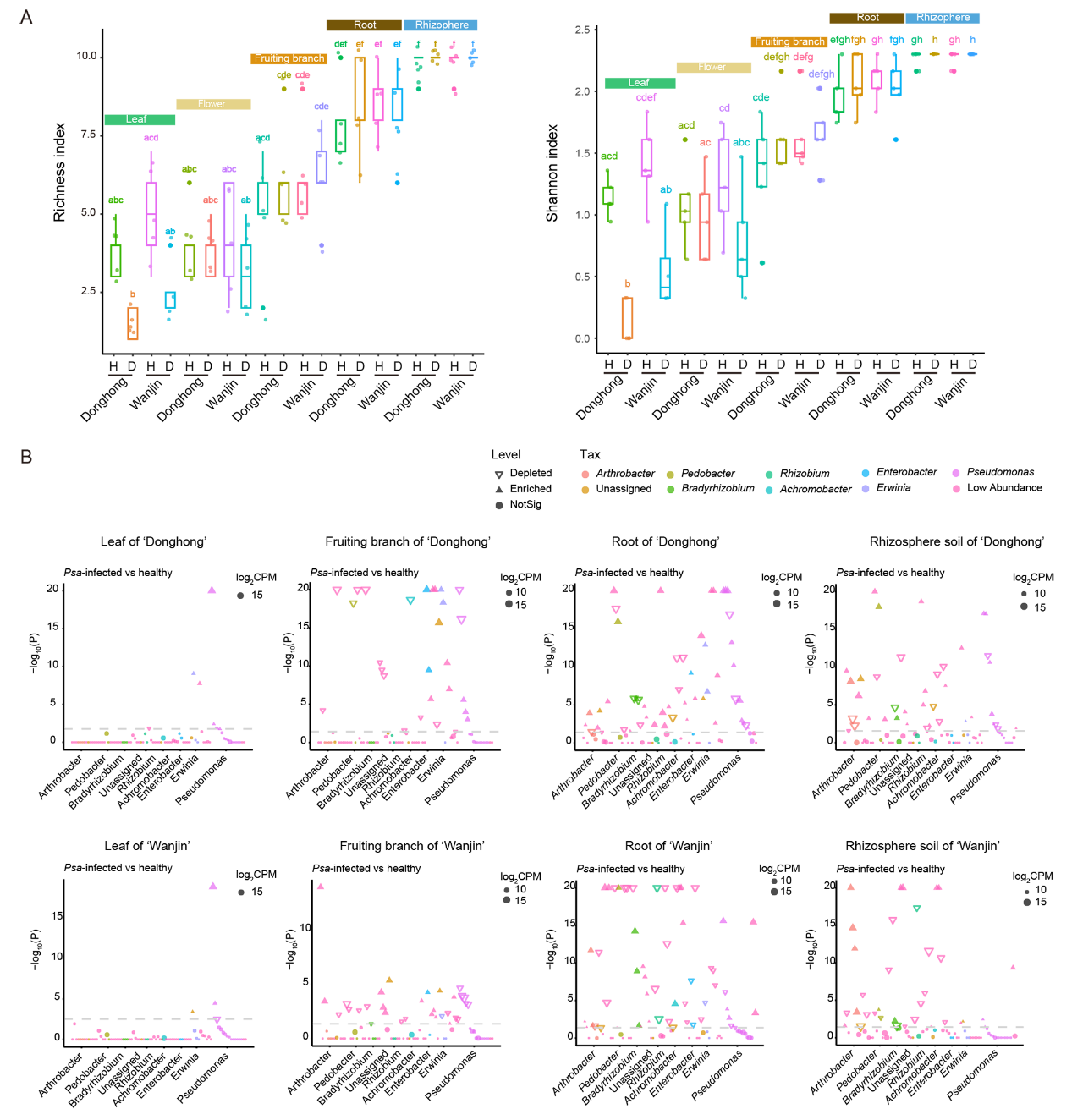


**Supplementary Figure 4: Effect of *Psa* infection on phyllosphere and rhizosphere associated bacteria of susceptible and resistant kiwifruit plants in Jinzhai County. A.** Richness index of bacteria associated with fruiting branches, roots, and rhizosphere of healthy and *Psa*-infected kiwifruit plants in Jinzhai County. **B**. Relative abundance of dominant genus in five niches of healthy and *Psa*-infected kiwifruit plants in Jinzhai county. **C**. Manhattan plots showing ASVs depleted or enriched in samples infected with *Psa* vs healthy samples in Jinzhai county. ASVs were arranged and colored according to the bacterial genus. The different letters represent significant differences (*p* < 0.05, ANOVA, Tukey HSD test).


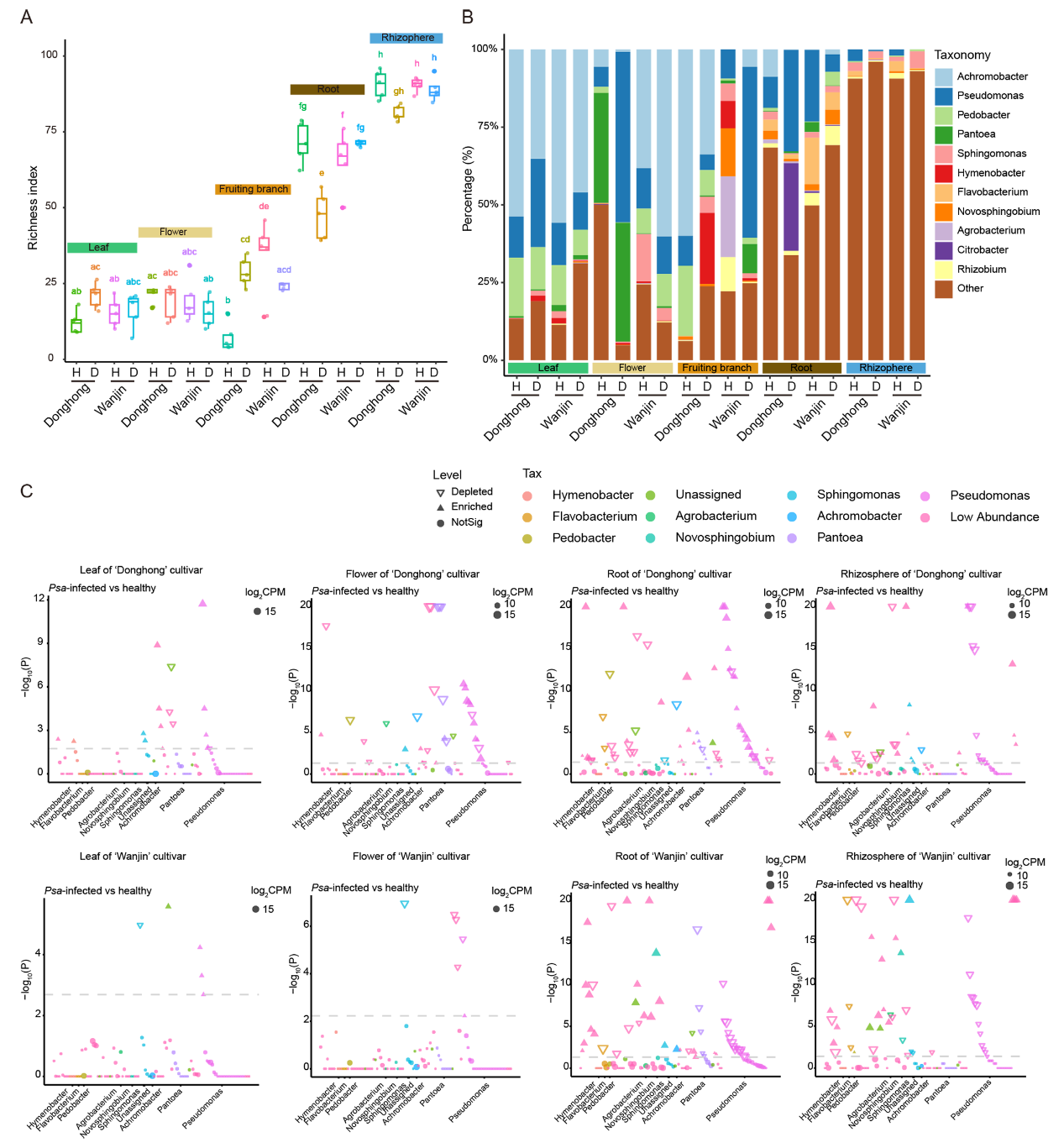


**Supplementary Figure 5: Maximum likelihood tree based on 16S rRNA sequences from 14 bacterial strains obtained in this study**


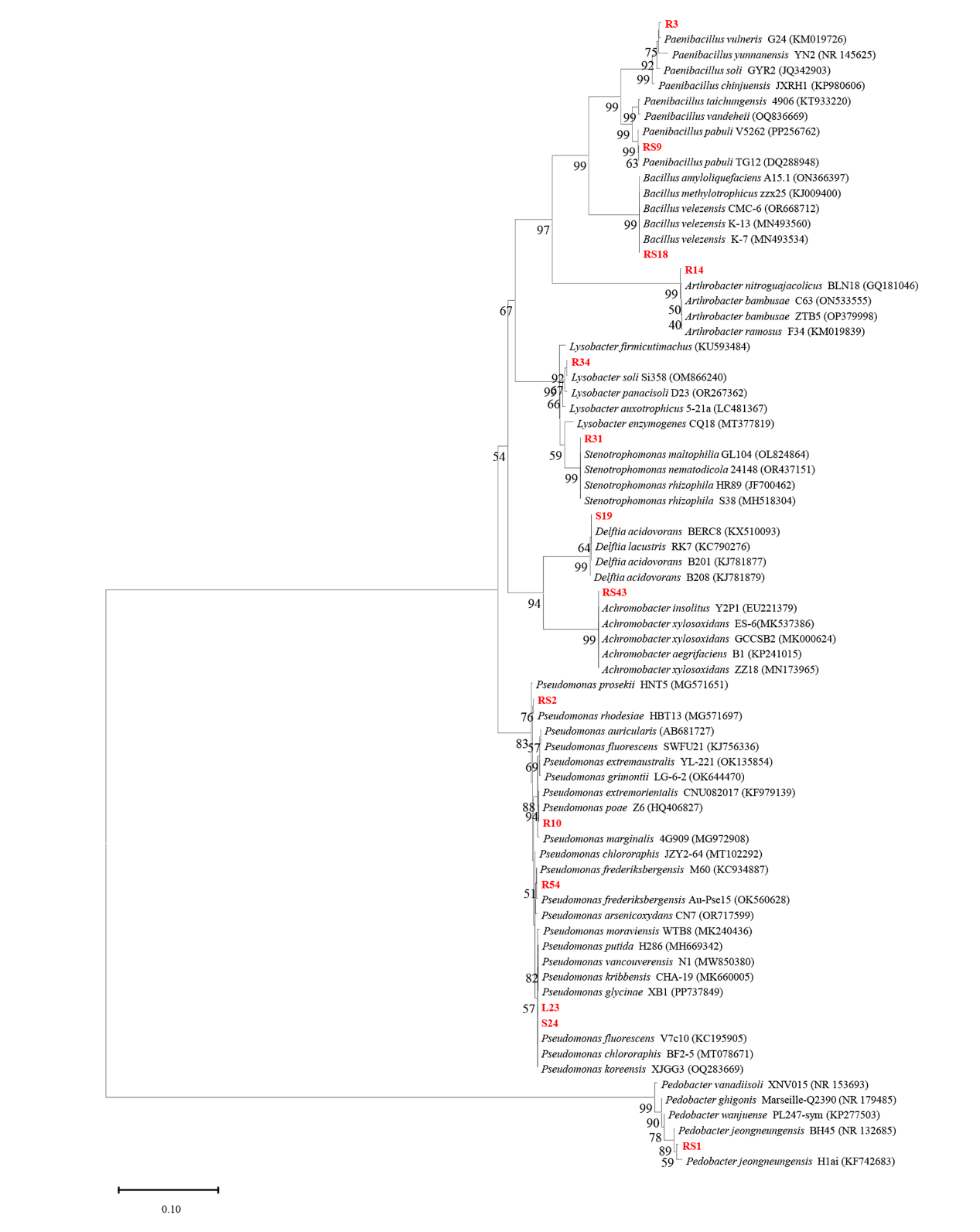


**Supplementary Figure 6:** **Compatibility between different biocontrol agents was tested by antagonistic activity. A**. R31 did not showed antagonistic activity against R10 on LB plate; **B**. R10 did not showed antagonistic activity against R31 on LB plate; **C**. R34 did not showed antagonistic activity against R10 on LB plate; **D**. R10 showed antagonistic activity against R34 on LB plate; **E**. R54 showed antagonistic activity against R10 on LB plate; **F**. R10 showed antagonistic activity against R54 on LB plate.


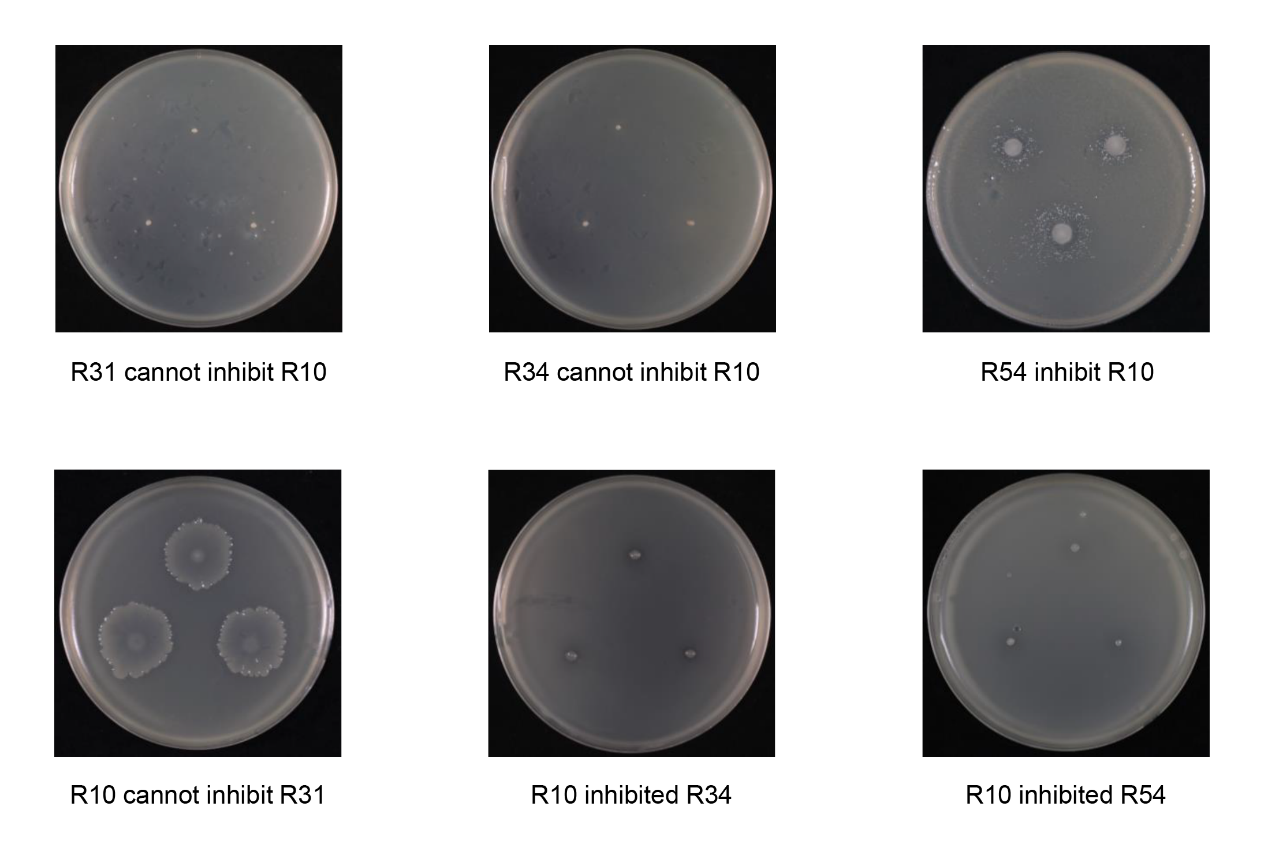

Supplement: Web_Material_uhae236 [file web_material_uhae236.zip › Supplementary Figures 1-6.docx]
